# Prognostic utility of exercise cardiovascular magnetic resonance in patients with systemic sclerosis-associated pulmonary arterial hypertension

**DOI:** 10.1093/ehjci/jeae177

**Published:** 2024-08-19

**Authors:** James T Brown, Ruta Virsinskaite, Tushar Kotecha, Jennifer A Steeden, Marianna Fontana, Nina Karia, Benjamin E Schreiber, Voon H Ong, Christopher P Denton, J Gerry Coghlan, Vivek Muthurangu, Daniel S Knight

**Affiliations:** National Pulmonary Hypertension Service, Royal Free London NHS Foundation Trust, Pond Street, London NW3 2QG, UK; Department of Cardiac MRI, Royal Free London NHS Foundation Trust, Pond Street, London NW3 2QG, UK; UCL Institute of Cardiovascular Science, University College London, Gower Street, London WC1E 6BT, UK; National Pulmonary Hypertension Service, Royal Free London NHS Foundation Trust, Pond Street, London NW3 2QG, UK; Department of Cardiac MRI, Royal Free London NHS Foundation Trust, Pond Street, London NW3 2QG, UK; UCL Institute of Cardiovascular Science, University College London, Gower Street, London WC1E 6BT, UK; National Pulmonary Hypertension Service, Royal Free London NHS Foundation Trust, Pond Street, London NW3 2QG, UK; Department of Cardiac MRI, Royal Free London NHS Foundation Trust, Pond Street, London NW3 2QG, UK; UCL Institute of Cardiovascular Science, University College London, Gower Street, London WC1E 6BT, UK; UCL Institute of Cardiovascular Science, University College London, Gower Street, London WC1E 6BT, UK; Department of Cardiac MRI, Royal Free London NHS Foundation Trust, Pond Street, London NW3 2QG, UK; UCL Division of Medicine, University College London, Royal Free Campus, Rowland Hill Street, London NW3 2PF, UK; National Pulmonary Hypertension Service, Royal Free London NHS Foundation Trust, Pond Street, London NW3 2QG, UK; Department of Cardiac MRI, Royal Free London NHS Foundation Trust, Pond Street, London NW3 2QG, UK; UCL Institute of Cardiovascular Science, University College London, Gower Street, London WC1E 6BT, UK; National Pulmonary Hypertension Service, Royal Free London NHS Foundation Trust, Pond Street, London NW3 2QG, UK; Centre for Rheumatology and Connective Tissue Diseases, UCL Medical School, Royal Free Campus, London, UK; Centre for Rheumatology and Connective Tissue Diseases, UCL Medical School, Royal Free Campus, London, UK; National Pulmonary Hypertension Service, Royal Free London NHS Foundation Trust, Pond Street, London NW3 2QG, UK; UCL Institute of Cardiovascular Science, University College London, Gower Street, London WC1E 6BT, UK; National Pulmonary Hypertension Service, Royal Free London NHS Foundation Trust, Pond Street, London NW3 2QG, UK; Department of Cardiac MRI, Royal Free London NHS Foundation Trust, Pond Street, London NW3 2QG, UK; UCL Institute of Cardiovascular Science, University College London, Gower Street, London WC1E 6BT, UK

**Keywords:** exercise, cardiopulmonary exercise testing, cardiovascular magnetic resonance, prognosis, pulmonary arterial hypertension, systemic sclerosis

## Abstract

**Aims:**

Systemic sclerosis complicated by pulmonary arterial hypertension (SSc-PAH) is a rare condition with poor prognosis. The majority of patients are categorized as intermediate risk of mortality. Cardiovascular magnetic resonance (CMR) is well placed to reproducibly assess right heart size and function, but most patients with SSc-PAH have less overtly abnormal right ventricles than other forms of PAH. The aim of this study was to assess if exercise CMR measures of cardiac size and function could better predict outcome in patients with intermediate risk SSc-PAH compared with resting CMR.

**Methods and results:**

Fifty patients with SSc-PAH categorized as intermediate risk underwent CMR-augmented cardiopulmonary exercise testing. Most patients had normal CMR-defined resting measures of right ventricular (RV) size and function. Nine (18%) patients died during a median follow-up period of 2.1 years (range 0.1–4.6). Peak exercise RV indexed end-systolic volume (ESVi) was the only CMR metric to predict prognosis on stepwise Cox regression analysis, with an optimal threshold < 39 mL/m^2^ to predict favourable outcome. Intermediate-low risk patients with peak RVESVi < 39 mL/m^2^ had significantly better survival than all other combinations of intermediate-low/-high risk status and peak RVESVi< or ≥39 mL/m^2^. In our cohort, ventilatory efficiency and resting oxygen consumption (VO_2_) were predictive of mortality, but not peak VO_2_, peak cardiac output, or peak tissue oxygen extraction.

**Conclusion:**

Exercise CMR assessment of RV size and function may help identify SSc-PAH patients with poorer prognosis amongst intermediate risk cohorts, even when resting CMR appears reassuring, and could offer added value to clinical PH risk stratification.

## Graphical Abstract

**Graphical Abstract:**
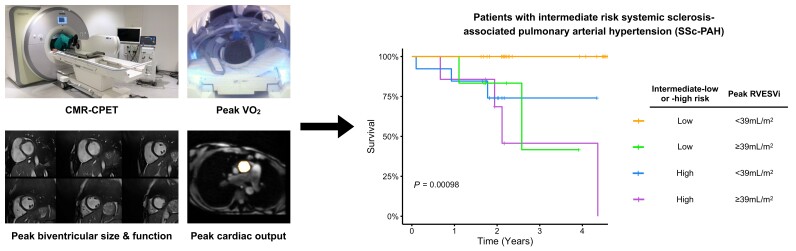

## Introduction

Cardiovascular magnetic resonance (CMR) is a key imaging modality in the assessment of pulmonary hypertension (PH). Metrics of right ventricular (RV) size and function by CMR are accurate, reproducible, and prognostic in PH.^1,2^ Consequently, CMR-derived parameters of RV size and function have been used to augment clinical PH risk stratification tools and as end-points in trials of targeted PH therapy.^3,4^ This is reflected by international guidelines that now incorporate CMR metrics into the comprehensive risk assessment of patients with pulmonary arterial hypertension (PAH).^5^ Cardiovascular MR is particularly useful in patients with systemic sclerosis-associated pulmonary arterial hypertension (SSc-PAH) where it also helps to identify additional relevant SSc-specific cardiovascular pathology.^6^

Evaluating disease severity through standardized risk of death assessment is pivotal to the clinical management of patients with PAH, facilitating a systematic approach to treatment escalation decisions as well as enabling prognostication.^5^ The majority of patients with PAH, including those with SSc-PAH, are classified as intermediate risk.^7,8^ In the 2022 European Society of Cardiology/European Respiratory Society (ESC/ERS) PH guidelines, this category is further subdivided into intermediate-low and intermediate-high risk strata based upon a composite score of functional class (FC), 6 min walk distance (6MWD) and N-terminal pro-B-type natriuretic peptide (NT-proBNP).^5^ This was done to provide better discrimination of risk and direction of treatment strategies during follow-up using routinely collected clinical data. However, the additional role of CMR is less clear in these two intermediate risk groups as they have less overtly abnormal right ventricles, particularly when compared with idiopathic PAH (IPAH).^9^ One potential solution could be exercise CMR (Ex-CMR), which has been used to unmask pathophysiology in a range of cardiovascular diseases and can predict outcome in patients with heart muscle disease.^10,11^ Recently, we have combined exercise CMR with cardiopulmonary exercise testing (CMR-CPET) to provide truly comprehensive evaluation of the determinants of exercise capacity. We have previously used CMR-CPET to better understand mechanisms of exercise intolerance in patients with SSc with and without PH^12^ and believe that it could be used to better evaluate risk in SSc-PAH. The aim of this study was to assess if Ex-CMR metrics are more predictive of outcome in patients with intermediate risk SSc-PAH compared with resting CMR.

## Methods

### Patient population

We performed a prospective, single-centre, observational study of 50 patients with a confirmed diagnosis of SSc-PAH categorized as intermediate risk who underwent CMR-CPET between March 2019 and March 2022. Patients were recruited from the National PH Service at the Royal Free Hospital, one of seven UK specialist centres for the management of adult PH. Inclusion criteria were: (i) a confirmed diagnosis of PAH by right heart catheterization according to contemporaneous international guidelines;^13^ (ii) diagnosis of SSc (including those with a concomitant overlap syndrome diagnosis) fulfilling the American College of Rheumatology/European League Against Rheumatism 2013 classification criteria;^14^ (iii) PH categorized as intermediate risk (either intermediate-low or intermediate-high) according to the 2022 ESC/ERS PH guidelines based upon the average risk score of NT-proBNP (obtained on the day of testing) and FC and 6MWD (both obtained at the last outpatient clinic appointment);^5^ and (iv) age 18–80 years. Exclusion criteria were (i) general contraindications to CMR scanning, (ii) contraindications to performing an exercise test [unstable symptoms, including angina, exertional syncope, World Health Organization (WHO) FC IV symptoms, and musculoskeletal disease preventing exercise], (iii) previous symptomatic ischaemic heart disease or moderate-to-severe valvular disease, (iv) changes in targeted PH therapy within 3 months, and (v) significant lung parenchymal disease that may confound CPET results, such as interstitial lung disease (significant being defined as >20% lung volume on computed tomography).

The study complies with the Declaration of Helsinki and was approved by national ethics committee (IRAS project ID 226101; REC reference 17/LO/1499, National Health Service Health Research Authority UK CRN 058274). All subjects provided written informed consent. The study is registered with ClinicalTrials.gov Protocol Registration and Results System (ClinicalTrials.gov ID: 100358).

### Clinical data

Patients were subdivided into intermediate-low and intermediate-high risk categories using the current ESC/ERS guidelines.^5^ Functional class and 6MWD were obtained from the most recent outpatient clinic encounter on the PH service database. Blood tests for full blood count and NT-proBNP were obtained on the day of CMR-CPET, prior to the exercise scan. The most recent lung function test data and the most recent cardiac catheterization data were obtained from patient electronic records systems and specialist services databases by clinicians blinded to the patient outcomes. Outcome was ascertained by checking the patient summary care record on the National Health Service (NHS) spine portal on 15 November 2023.

### CMR-augmented cardiopulmonary exercise testing

Cardiovascular magnetic resonance-augmented CPET was performed as previously described.^12^ Imaging was performed on a 1.5 T CMR scanner (Magnetom Aera, Siemens, Erlangen, Germany) using two six-element coils (one spinal matrix, one body matrix). The scanning room was temperature controlled. Full resuscitation facilities were available. Each subject’s electrocardiogram was monitored continuously using the in-built system in the CMR scanner. This system allowed assessment of rate and rhythm but is not suitable for identification of ischaemia. All patients had peripheral venous access during testing for use in resuscitation protocols in the event of clinical instability.

### CMR imaging techniques (real-time flow and volume imaging)

Before exercise, subjects underwent a routine CMR with long- and short-axis cine imaging, myocardial native T1 and T2 mapping as previously described.^12,15^ At rest and peak exercise, aortic flow was measured using real-time phase-contrast (PC) CMR (PC-CMR). PC-CMR was performed using a uniform density golden-angle spiral sequence, with a compressive sensing (CS) reconstruction.^16^ Real-time assessment of biventricular volumes was performed at rest and peak exercise, immediately after each real-time flow acquisition using a 2D multi-slice real-time tiny golden-angle spiral CS balanced steady state free precession sequence.^17^ All real-time imaging was acquired during free breathing with parameters as previously described.^12^ The aortic flow and short-axis image data were reconstructed off-line (MATLAB R2018a, MathWorks Inc., Natick, MA, USA), using the Berkeley Advanced Reconstruction Toolbox (BART).^12^

### Respiratory gas analysis

Breath-by-breath gas exchange analysis was performed using a commercial CPET system (Ultima, MedGraphics, St Paul, MN, USA). The analyser was placed in the CMR control room and attached to the facemask (Hans Rudolph, Kansas City, USA) via a set of CMR-compatible sampling tubes (umbilicus) passed through the waveguide. This bespoke umbilicus was modified as previously described increasing overall length from the standard 234–1000 cm and removing ferromagnetic components.^18^ It was thoroughly tested by the manufacturer, meeting all quality control standards. Gas and flow calibrations were performed before each test and at least 30 min after system initiation. All measurements were taken at body temperature and ambient pressure.

### Exercise protocol

Subjects performed exercise on a supine CMR-compatible cycle ergometer (MR Cardiac Ergometer Pedal, Lode, Groningen, Netherlands) as previously described.^12^ The first minute of the protocol consisted of exercise against zero resistance, with subjects asked to cycle at 60–70 rpm. Thereafter, the protocol was split in 2 min stages, each containing three workload increments introduced at 0, 30, and 60 s into the stage as follows—stages 1–3: 3 Watts (W), stages 4–6: 5 W, stages 7–8: 7 W, stages 9–10: 9 W, and stages 11–12: 11 W. The smaller increments at the start of the protocol ensured that even subjects with significant exercise intolerance were able to complete at least two exercise stages. This protocol was followed until exhaustion. At the onset of exhaustion (defined as an inability to maintain cadence or a verbal indication from the subject), the subject was encouraged to maintain cycling while peak aortic flow and ventricular volumes were acquired, after which exercise was stopped.

### Data analysis

All CMR studies were reported by experienced clinical CMR observers (J.T.B., D.S.K.). blinded to patient outcomes and analysed using ‘in-house’ plug-ins for OsiriX MD version 9.0.1 (Pixmeo Sarl, Bernex, Switzerland).^19–21^

PC-CMR flow data of the ascending thoracic aorta were segmented using a semi-automatic vessel edge detection algorithm with manual operator correction if required. Stroke volume (SV) was calculated by integrating the flow curve across a single R–R interval. Cardiac output (CO) was given by SV × heart rate (HR). End-diastole and end-systole were defined per short-axis slice by visual assessment of cavity areas and segmentation was performed manually. Papillary muscles and trabeculae were excluded from the blood pool. Left and right ventricular (LV and RV) stroke volumes were calculated as the difference between the end-diastolic volume (EDV) and end-systolic volume (ESV). Ejection fraction (EF) was determined as (SV/EDV) × 100. Bi-atrial areas were traced at end-systole on a four-chamber cine image acquired at rest. All volumetric data, area data, and CO were indexed to body surface area (BSA) and denoted by the suffix -i. Native myocardial T1 and T2 relaxation times were measured by drawing a region of interest (ROI) within the interventricular septum on the mid-cavity short-axis maps remote from the insertion points.^15^

Oxygen uptake (VO_2_) and respiratory exchange ratio (RER) measurements were time-registered to CMR data. The VO_2_ was indexed to body weight and denoted by the prefix -i. Arteriovenous oxygen content gradient was calculated as ΔavO_2_ = VO_2_/CO (using non-indexed data). These calculations were performed at rest and peak exercise for all subjects. Ventilatory efficiency was evaluated as the slope of the minute ventilation carbon dioxide production (VE/VCO_2_) plot in the portion where there was a linear relationship.

### Statistics

All statistical testing was performed using R (RStudio 2021.09.2 using R 4.1.2). Data were examined for normality using the Shapiro–Wilk normality test. Descriptive statistics were expressed as mean (± standard deviation) for normally distributed data and median (interquartile range) for non-normally distributed data. Differences between dead and alive groups were performed using *t*-tests (normal data) and Mann–Whitney tests (non-normal data). Univariable survival analysis was performed using Cox Proportional Hazards regression with hazard ratio (HR) reported per standard deviation change in continuous metrics. Due to collinearity and the small population size, bidirectional stepwise Cox regression was used to select the most parsimonious model with all significant CMR metrics from univariable analysis being entered. Optimal cut-offs for prognostic metrics from stepwise Cox were identified using maximally selected rank statistics. These defined reduced and normal RV contractile reserve groups that were then additionally combined with intermediate-low and intermediate-high risk groups. Survival differences were assessed using Kaplan–Meier plots with both omnibus and pairwise Log-Rank tests (with adjustment for multiple comparisons performed using the Benjamini and Hochberg method). For all tests, a *P* value < 0.05 was considered statistically significant.

## Results

### Study population

Clinical details of the study population and targeted PH therapies are shown in *Table 1*. The median age was 65 (full range 29–76) years and patients were predominantly female (48, 96%). As expected, the majority of patients had limited (44, 88%) rather than diffuse (6, 12%) SSc disease. Four (8%) patients had an overlap connective tissue disease diagnosis. There were 30 (60%) patients in the intermediate-low risk category and 20 (40%) in the intermediate-high risk category. The majority of patients (44, 88%) was prevalent for their PAH diagnosis. Nine (18%) patients died during a median follow-up period of 2.1 years (range 0.1–4.6 years). Compared with survivors, patients who died during follow-up had significantly higher NT-proBNP (*P* = 0.033), but similar 6MWD (*P* = 0.70) and FC (*P* = 0.77).

**Table 1:** Clinical details of the study cohort

	SSc-PAH patients (*n* = 50)
Demographics and co-morbidities	
Female, *n* (%)	48 (96)
Age [median (full range)], years	65 (29–76)
Incident/prevalent PAH diagnosis, *n* (%)	6/44 (12/88)
Intermediate-low/intermediate-high risk, *n* (%)	30 (60)/20 (40)
Background haemodynamic and clinical measurements	
PVR (dynes/s/cm^−5^)	291 (214–400)
mPAP (mmHg)	31 (24–36)
FVC (%predicted)	94.9 ± 18.9
FEV1 (%predicted)	86.7 ± 16.4
DLCO (%predicted)	40.6 ± 10.2
Targeted PH therapies (*n* = 44, 88%)	
PDE5I, *n* (%)	39 (78)
ERA, *n* (%)	37 (74)
SGCS, *n* (%)	2 (4)
Prostanoids (IV/oral IP receptor agonist), *n* (%)	1 (2)/4 (8)
SSc details	
Limited/diffuse SSc, *n* (%)	44/6 (88/12)
Overlap syndromes, *n* (%)	4 (8)
Myositis, *n* (%)	2 (4)
SLE, *n* (%)	2 (4)

Normally distributed data displayed as mean ± SD. Non-normally distributed data shown as median (interquartile range) unless otherwise specified.

DLCO, diffusion capacity of the lung for carbon monoxide; ERA, endothelin receptor antagonist; FEV1, forced expiratory volume in 1 s; FVC, forced vital capacity; IV, intravenous; mPAP, mean pulmonary arterial pressure; PAH, pulmonary arterial hypertension; PDE5I, phosphodiesterase type 5 inhibitor; PVR, pulmonary vascular resistance; SGCS, soluble guanylate cyclase stimulator; SLE, systemic lupus erythematosus; SSc, scleroderma/systemic sclerosis.

### Resting CMR-CPET

Resting CMR-CPET data are shown in *Table 2*. Compared with published normal values,^22^ 8% of patients had abnormal RVEDVi, 24% had abnormal RVESVi, and 36% had abnormal RVEF. The only significant differences in resting CMR metrics between dead and alive patients were indexed right atrial area (RAi, *P* = 0.034) and septal T2 (*P* = 0.040), which were both higher in patients who died during follow-up. Importantly, there were no significant differences in resting biventricular size or function between dead and alive patients. Patients who died during follow-up also had higher resting VO_2_ (*P* = 0.040).

**Table 2:** Differences in clinical data, PAH risk stratification data, and resting CMR-CPET data in dead compared with alive patients

	Total cohort (*n* = 50)	Alive (*n* = 41)	Dead (*n* = 9)	*P* value
Clinical data and PAH risk stratification data				
Age (years)	65 (58–71)	64 (58–69)	69 (65–71)	0.18
6MWD (m)	361 ± 99	363 ± 103	349 ± 82	0.70
NT-proBNP (ng/L)	378 (154–685)	256 (127–616)	1590 (207–2230)	0.033^a^
WHO FC	3 (2–3)	3 (2–3)	3 (2–3)	0.77
PAH risk score	2 (2–3)	2 (2–3)	3 (3–3)	0.011^a^
Hb (g/L)	120 (113–129)	120 (111–129)	119 (117–122)	0.88
Resting CMR metrics				
RVEDVi (mL/m^2^)	75 ± 18	73 ± 17	82 ± 17	0.16
RVESVi (mL/m^2^)	29 (22–41)	28 (22–36)	44 (34–46)	0.098
RVSVi (mL/m^2^)	41 ± 11	41 ± 11	44 ± 8	0.48
RVEF (%)	56 ± 11	57 ± 11	54 ± 10	0.56
LVEDVi (mL/m^2^)	65 ± 15	65 ± 16	67 ± 13	0.77
LVESVi (mL/m^2^)	23 (16–28)	22 (16–28)	23 (14–31)	0.85
LVSVi (mL/m^2^)	42 ± 11	41 ± 12	44 ± 7	0.60
LVEF (mL/m^2^)	66 (59–72)	66 (58–72)	68 (62–76)	0.54
HR (bpm)	74 ± 12	74 ± 13	74 ± 9	0.99
COi (L/min/m^2^)	3.1 ± 0.8	3.0 ± 0.9	3.3 ± 0.6	0.41
RAi area (cm^2^/m^2^)	13 ± 3	13 ± 3	15 ± 4	0.034^a^
LAi area (cm^2^/m^2^)	14 ± 3	13 ± 3	15 ± 3	0.23
Native myocardial T1 (ms)	1082 ± 55	1078 ± 53	1096 ± 63	0.37
Myocardial T2 (ms)	52 ± 4	52 ± 4	55 ± 5	0.04^a^
Resting CPET metrics				
iVO_2_ (mL/min/kg)	3.4 (3.0–4.0)	3.2 (3.0–4.0)	3.8 (3.5–5.0)	0.040^a^
avO_2_ (mLO_2_/100 mL)	4.2 (3.9–5.0)	4.2 (3.9–5.0)	4.2 (3.9–4.3)	0.85

Normally distributed data displayed as mean ± SD. Non-normally distributed data shown as median (interquartile range) unless otherwise specified.

6MWD, 6 min walk distance; iVO_2_, oxygen consumption indexed to weight; avO_2_, tissue oxygen extraction; CMR, cardiovascular magnetic resonance; COi, cardiac output indexed to BSA; CPET, cardiopulmonary exercise test; FC, functional class; Hb, haemoglobin; HR, heart rate; LAi, left atrial area indexed to body surface area (BSA); NT-proBNP, N-terminal pro-B-type natriuretic peptide; PAH, pulmonary arterial hypertension; RAi, right atrial area indexed to BSA; RVEDVi, BSA-indexed right ventricular end-diastolic volume; RVESVi, BSA-indexed right ventricular end-systolic volume; RVSVi, BSA-indexed right ventricular stroke volume; RVEF, right ventricular ejection fraction; LVEDVi/LVESVi/LVSVi/LVEF, left ventricular measurements as per RV; WHO, World Health Organization.

^a^Statistically significant with two-sided *P* < 0.05.

### Exercise feasibility

All subjects successfully completed the exercise protocol without complication; no subjects required medical intervention. Peak-exercise RER ≥1.0 was achieved in 44 (88%) patients; of the six patients who exercised to exhaustion but did not reach anaerobic threshold, the lowest peak RER was 0.88. Changes in CMR-CPET metrics with exercise are shown in Supplementary data online, *Table S1*. There were significant decreases of RVEDV (*P* = 0.014), LVEDV (*P* < 0.001), and LVESV (*P* < 0.001) on exercise along with significant increases in LVEF (*P* = 0.034), COi (*P* < 0.001), iVO_2_ (*P* < 0.001), and ΔavO_2_ (*P* < 0.001). There was no significant difference in RVEF between rest and peak exercise (*P* = 0.36).

The only significant difference in either rest or exercise CMR metrics between the intermediate-low risk and intermediate-high risk groups was exercise RVESV, which was lower in intermediate-low risk patients (*P* = 0.043, Supplementary data online, *Table S2*). Differences in exercise CMR-CPET metrics in dead compared with alive patients are shown in *Table 3*. The only significant differences were higher peak RVESVi (*P* = 0.017), lower peak RVEF (*P* = 0.044), and higher VE/VCO_2_ (*P* = 0.011) in patients who died during follow-up.

**Table 3:** Differences in exercise CMR-CPET metrics in dead compared with alive patients

	Total cohort (*n* = 50)	Alive (*n* = 41)	Dead (*n* = 9)	*P* value
Exercise CMR metrics				
RVEDVi (mL/m^2^)	69 ± 17	68 ± 17	76 ± 17	0.17
RVESVi (mL/m^2^)	26 (19–39)	23 (18–36)	44 (27–45)	0.017^a^
RVSVi (mL/m^2^)	37 (32–44)	39 (32–44)	35 (33–37)	0.57
RVEF (%)	58 ± 14	59 ± 14	49 ± 8	0.044^a^
LVEDVi (mL/m^2^)	58 ± 14	59 ± 15	55 ± 13	0.48
LVESVi (mL/m^2^)	16 (12–24)	16 (12–24)	14 (11–23)	0.70
LVSVi (mL/m^2^)	39 ± 12	40 ± 12	36 ± 8	0.42
LVEF (mL/m^2^)	73 (60–77)	73 (65–77)	76 (59–77)	0.79
HR (bpm)	108 ± 15	108 ± 16	104 ± 10	0.47
COi (L/min/m^2^)	4.1 ± 1.1	4.2 ± 1.2	3.6 ± 0.6	0.16
Exercise CPET metrics				
iVO_2_ (mL/min/kg)	9.7 ± 1.8	9.9 ± 1.9	9.0 ± 1.5	0.20
avO_2_ (mLO_2_/100 mL)	8.7 (7.5–10.9)	8.7 (7.5–11.3)	9.0 (7.5–10.2)	0.85
VE/VCO_2_	45.2 ± 10.2	43.5 ± 10.0	52.8 ± 7.7	0.011^a^

Normally distributed data displayed as mean ± SD. Non-normally distributed data shown as median (interquartile range) unless otherwise specified.

iVO_2_, oxygen consumption indexed to weight; avO_2_, tissue oxygen extraction; CMR, cardiovascular magnetic resonance; COi, cardiac output indexed to BSA; CPET, cardiopulmonary exercise test; HR, heart rate; RVEDVi, BSA-indexed right ventricular end-diastolic volume; RVESVi, BSA-indexed right ventricular end-systolic volume; RVSVi, BSA-indexed right ventricular stroke volume; RVEF, right ventricular ejection fraction; LVEDVi/LVESVi/LVSVi/LVEF, left ventricular measurements as per RV; VE/VCO_2_, ventilatory equivalents for carbon dioxide.

^a^Statistically significant with two-sided *P* < 0.05.

### Predictors of mortality

**Figure 1:**
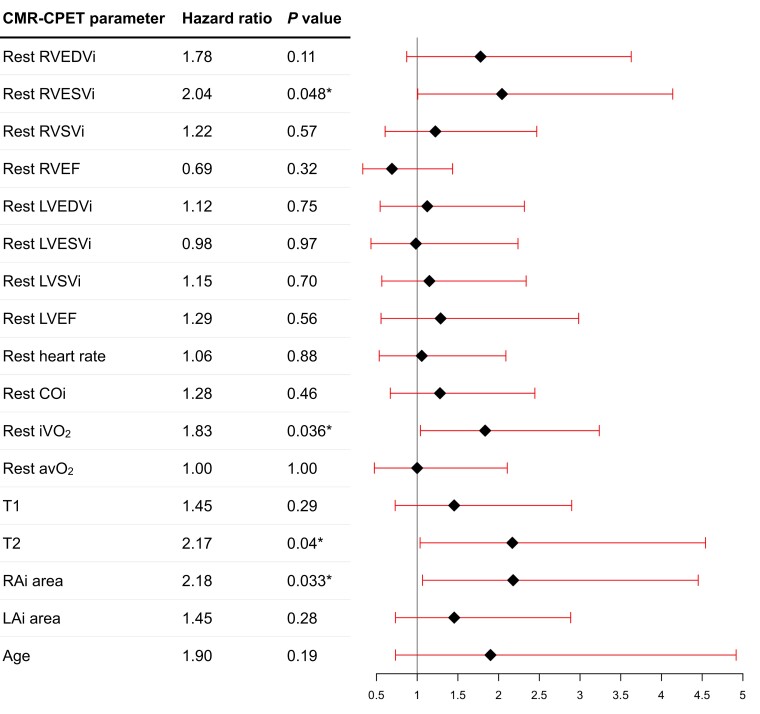
Forest plot of resting CMR and CPET variables to predict all-cause mortality on univariable Cox regression analysis. Asterisk denotes statistically significant metrics (*P* < 0.05).

**Figure 2:**
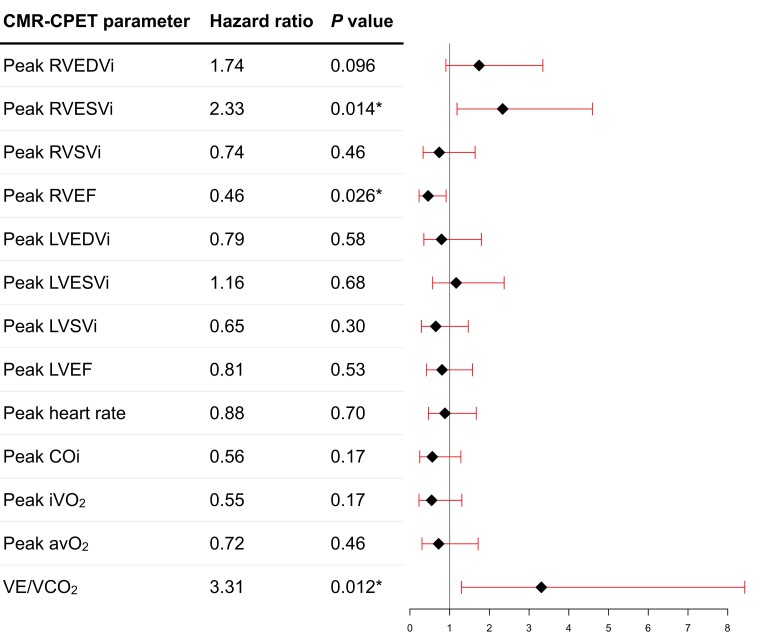
Forest plot of peak exercise CMR and CPET variables to predict all-cause mortality on univariable Cox regression analysis. Asterisk denotes statistically significant metrics (*P* < 0.05).

**Figure 3:**
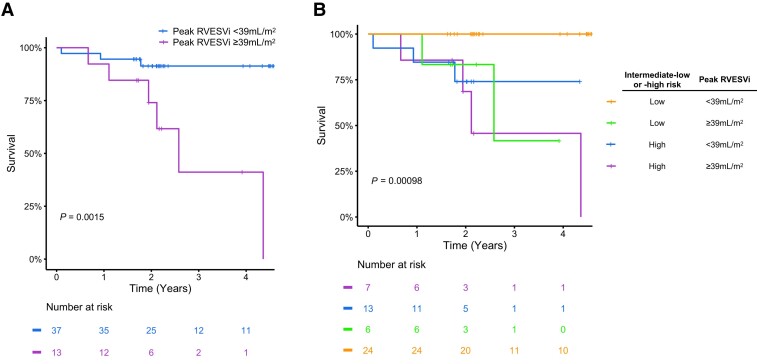
Kaplan–Meier plots showing: (*A*) survival curves for reduced and normal right ventricular contractile reserve groups, as defined by a peak RVESVi threshold of <39 mL/m^2^; (*B*) survival curves for four groups defined by all combinations of reduced and normal RV contractile reserve and intermediate-low/-high risk.

Resting univariable CMR predictors of all-cause mortality (*Figure 1*) included RVESVi (*P* = 0.048), myocardial T2 (*P* = 0.040), and RAi (*P* = 0.033). Peak Ex-CMR predictors of all-cause mortality (*Figure 2*) included RVESVi (*P* = 0.014) and RVEF (*P* = 0.026). Neither rest nor exercise CMR metrics of LV size and function was prognostic in our study cohort. On stepwise analysis including all these variables, only peak RVESVi (*P* = 0.039) was selected. The optimal threshold of peak RVESVi was <39 mL/m^2^ (*P* = 0.0015) and dichotomized survival curves based on this cut-off show clear separation (*Figure 3A*). When combined with intermediate-low or intermediate-high risk status, intermediate-low risk patients with peak RVESVi < 39 mL/m^2^ had significantly better survival than all other groups (*Figure 3B*), including intermediate-low risk patients with peak RVESVi ≥ 39 mL/m^2^ (*P* = 0.0067). There were no significant survival differences between the other three groups.

## Discussion

The main findings of this CMR-CPET study of a cohort of intermediate risk patients with SSc-PAH were: (i) the majority of patients had normal CMR-defined resting measures of RV size and function; (ii) peak RVESVi was the only CMR metric to predict prognosis on stepwise Cox regression analysis, with an optimal threshold < 39 mL/m^2^ to predict a favourable outcome; (iii) intermediate-low risk patients with peak RVESVi < 39 mL/m^2^ had significantly better survival than all other combinations of intermediate-low/-high risk status and peak RVESVi; and (iv) VE/VCO_2_ and resting VO_2_ were predictive of all-cause mortality in this cohort, but not peak VO_2_, peak COi or peak ΔavO_2_. Although Ex-CMR has been shown to be predictive of cardiac decompensation and arrhythmia in dilated cardiomyopathy,^11^ to our knowledge, this is the first mortality-driven study demonstrating the prognostic capacity of Ex-CMR.

It is well known that the response of the RV to elevated afterload is the major determinant of outcome in PAH,^23^ hence CMR is well placed to aid risk stratification in these patients. However, the majority of studies demonstrating the relationship between resting CMR-derived RV metrics and survival in PAH included sicker patients, as evidenced by more severely deranged RV function.^1,2^ Thus, the role of CMR in risk stratifying patients with less abnormal RV function, such as in our cohort, is not as well defined. This is particularly pertinent in patients with SSc-PAH, who tend to have more favourable pulmonary haemodynamics and RV function^9^ as well as being more likely to be classified as intermediate risk.^7,8^ In fact, resting RVESVi was only just predictive of prognosis in this study, suggesting that resting CMR metrics may be less sensitive in this important sub-population. Conversely, both peak exercise RVEF and RVESVi were strongly predictive of all-cause mortality.

Additionally, peak RVESVi < 39 mL/m^2^ combined with intermediate-low risk status identified a subgroup of patients with significantly better prognosis. Thus, while resting RVESV has been shown to augment clinically utilized risk score calculators,^3^ our results show that RV contractile reserve might better predict outcome in intermediate risk SSc-PAH patients. This is analogous to LV contractile reserve (measured using echocardiography with either pharmacological or exercise stressors) predicting outcome in non-ischaemic heart muscle disease.^24^ However, robust non-invasive assessment of RV contractile reserve requires reliable and reproducible measures that we believe only CMR can currently provide. Furthermore, exercise stress is preferable to pharmacological agents, both because it is more physiological and causes less side-effects. This finding, if corroborated by future studies, would have some implications for risk stratification, which is central to treatment choices and management strategies in PAH.^5^ Current metrics such as FC, 6MWD, and BNP enable quick risk stratification in PAH patients in the outpatient clinic setting. However, our results suggest that intermediate-low risk patients (defined by FC, 6MWD, and BNP) with peak RVESVi ≥ 39 mL/m^2^ have similar outcomes to intermediate-high risk patients. Furthermore, there was no mortality in intermediate-low risk patients with peak RVESVi < 39 mL/m^2^. Thus, inclusion of peak RVESVi allows the intermediate-low risk group to be subdivided in a prognostically important way, demonstrating the additive value of combining Ex-CMR with clinically derived risk strata.

Interestingly, we found that VE/VCO_2_ slope and resting VO_2_ were predictive of all-cause mortality in SSc-PAH stratified as intermediate risk. However, neither peak VO_2_, peak COi, nor peak ΔavO_2_ was prognostic. Resting VO_2_ reflects basal metabolic rate that is increased in patients with congestive heart failure and is also independently related to mortality.^25,26^ Thus, it is not surprising that this is predictive of outcome in our population. Similarly, VE/VCO_2_ has been shown to be prognostic in IPAH, although its predictive utility is less clear in studies of patients with PAH with associated conditions (APAH), such as SSc.^27,28^ The poor predictive capabilities of peak VO_2_, COi, and ΔavO_2_ may be surprising as these are well-established markers of cardiovascular fitness, and are known to be severely reduced in SSc-PAH.^12^ However, peak VO_2_ has previously been shown to not be predictive of outcome in study cohorts including patients with APAH.^27,29^ Thus, our study supports the suggestion that CPET-derived metrics might be more variable in APAH subcategories, particularly in multisystem disorders such as SSc, and require further dedicated evaluation. This also highlights the utility of Ex-CMR to directly evaluate the response of the RV to exercise in these patients.

### Limitations

Although 50 patients with confirmed SSc-PAH stratified as intermediate risk is a reasonable sized cohort relative to the rarity of the condition, this is nevertheless a single-centre study of a small patient cohort. Additionally, the optimal threshold for peak exercise RVESVi to predict outcome is specific to the CMR-CPET protocol that we used. Thus, future studies should be directed at developing reproducible protocols to aid dissemination to other centres and increasing the study population size. This would help validate our findings of additive prognostic benefit, and thus incorporation into routine SSc-PAH management. Achieving true peak exercise using a supine bicycle ergometer is also challenging and not directly comparable with conventional CPET. However, the majority of patients reached RER and we have previously demonstrated good correlation between CMR-CPET and conventional CPET in achieving peak iVO_2_.^18^ Another limitation of this study was the lack of contemporaneous invasive pressure data with the median time between the last catheterization and Ex-CMR being 426 days. This meant that risk stratification based on invasive pressures could not be performed and prevented comparison of the prognostic utility of exercise RV data compared with resting invasive haemodynamics. One possible solution is to leverage CMR-guided right heart catheterization, which is increasingly becoming feasible in the clinical environment in patients with PAH.^30^ In patients undergoing this procedure, it would be relatively easy to add an Ex-CMR study, enabling contemporaneous invasive pressures to be fully integrated into risk models. Furthermore, it would also be possible to measure stress invasive haemodynamics, which could provide even more granular prognostic information.

## Conclusions

Peak exercise assessment of RV size and function may help identify patients with poorer prognosis amongst intermediate risk cohorts of patients with SSc-PAH, even when resting CMR measurements and conventional clinical risk stratification appear relatively reassuring. Thus, Ex-CMR could offer added value to clinical PH risk stratification in SSc-PAH, particularly in intermediate risk patients, and may provide an additional investigative strategy when considering optimization of targeted PH therapies.

## Supplementary data

Supplementary data are available at *European Heart Journal - Cardiovascular Imaging* online.

## Supplementary Material

jeae177_Supplementary_Data

## Data Availability

The study dataset will be made available to other researchers for purposes of reproducing the results or replicating the procedure upon reasonable request to the corresponding author, subject to institutional and ethical committee approvals.
