# Supplementary material for: Prognostic utility of exercise cardiovascular magnetic resonance in patients with systemic sclerosis-associated pulmonary arterial hypertension
Source: Eur Heart J Cardiovasc Imaging. 2024 Aug 19;25(12):1712–20. doi: 10.1093/ehjci/jeae177 (PMC11601748; doi:10.1093/ehjci/jeae177)
Supplement: jeae177_Supplementary_Data [file jeae177_supplementary_data.zip › Supplementary data.docx]

**Supplementary Material**

**Prognostic utility of exercise CMR in patients with systemic sclerosis-associated pulmonary arterial hypertension.**

James T. Brown, MB BChir, Ruta Virsinskaite, MD, Tushar Kotecha, MBChB, PhD, Jennifer A. Steeden, PhD, Marianna Fontana, MD, PhD, Nina Karia, MBBS, Benjamin E. Schreiber, MD, MA, Voon H. Ong, PhD, Christopher P. Denton, PhD, J. Gerry Coghlan, MD, Vivek Muthurangu, MD,^*^ Daniel S. Knight, MBBS, MD(Res)^*^

* These authors contributed equally to the manuscript and should be considered joint last authors.

**Supplementary Material**

**Supplementary Table 1**……………………………………………………………….Page 3

**Supplementary Table 2**……………………………………………………………….Page 4

**Supplementary Table 1:** Changes in CMR-CPET metrics with exercise.

| **Variable** | **Rest** | **Exercise** | ***P* value** |
| --- | --- | --- | --- |
| **CMR metrics** | | | |
| RVEDVi (mL/m^2^) | 75 (44-124) | 68 (40-118) | 0.014* |
| RVESVi (mL/m^2^) | 32±12 | 30±14 | 0.11 |
| RVSVi (mL/m^2^) | 41±11 | 39±12 | 0.10 |
| RVEF (%) | 56±11 | 58±14 | 0.36 |
| LVEDVi (mL/m^2^) | 65±15 | 58±14 | <0.001* |
| LVESVi (mL/m^2^) | 23±9 | 19±9 | <0.001* |
| LVSVi (mL/m^2^) | 42±11 | 39±12 | 0.061 |
| LVEF (%) | 65±10 | 68±12 | 0.034* |
| COi (L/min/m^2^) | 3.1±0.8 | 4.1±1.1 | <0.001* |
| **CPET metrics** | | | |
| iVO_2_ (mL/min/kg) | 3.7±0.9 | 9.7±1.8 | <0.001* |
| avO_2_ (mLO_2_/100mL) | 4.2 (3.0-9.7) | 8.7 (5.9-19.5) | <0.001* |

Normally distributed data displayed as mean ± SD. Non-normally distributed data shown as median (interquartile range) unless otherwise specified.

*Statistically significant with two-sided *P* <0.05.

iVO_2_, oxygen consumption indexed to weight; avO_2_, tissue oxygen extraction; CMR, cardiovascular magnetic resonance; COi, cardiac output indexed to BSA; CPET, cardiopulmonary exercise test; RVEDVi, body surface area (BSA)-indexed right ventricular end-diastolic volume; RVESVi, BSA-indexed right ventricular end-systolic volume; RVSVi, BSA-indexed right ventricular stroke volume; RVEF, right ventricular ejection fraction; LVEDVi/LVESVi/LVSVi/LVEF, left ventricular measurements as per RV.

**Supplementary Table 2:** Differences between intermediate-low risk and intermediate-high risk patients in rest and exercise CMR metrics.

|  | **Total cohort (n = 50)** | **Intermediate-low risk (n = 30)** | **Intermediate-high risk (n = 20)** | ***P* value** |
| --- | --- | --- | --- | --- |
| Age (years) | 65 (58-71) | 65 (59-69) | 67 (58-72) | 0.43 |
| **Resting CMR metrics** | | | | |
| RVEDVi (mL/m^2^) | 75±18 | 72±18 | 78±17 | 0.27 |
| RVESVi (mL/m^2^) | 29 (22-41) | 27 (21-36) | 35 (26-44) | 0.13 |
| RVSVi (mL/m^2^) | 41±11 | 40±10 | 43±12 | 0.48 |
| RVEF (%) | 56±11 | 57±10 | 55±12 | 0.59 |
| LVEDVi (mL/m^2^) | 65±15 | 64±13 | 67±18 | 0.52 |
| LVESVi (mL/m^2^) | 23 (16-28) | 21 (16-28) | 23 (18-29) | 0.62 |
| LVSVi (mL/m^2^) | 42±11 | 41±10 | 43±13 | 0.68 |
| LVEF (mL/m^2^) | 66 (59-72) | 66 (60-71) | 66 (56-72) | 0.63 |
| **Exercise CMR metrics** | | | | |
| RVEDVi (mL/m^2^) | 69±17 | 67±18 | 73±16 | 0.23 |
| RVESVi (mL/m^2^) | 26 (19-39) | 22 (18-35) | 37 (23-43) | 0.043* |
| RVSVi (mL/m^2^) | 37 (32-44) | 40 (32-46) | 36 (33-39) | 0.46 |
| RVEF (%) | 58±14 | 61±14 | 53±14 | 0.071 |
| LVEDVi (mL/m^2^) | 58±14 | 57±12 | 59±18 | 0.79 |
| LVESVi (mL/m^2^) | 16 (12-24) | 14 (12-21) | 19 (12-29) | 0.46 |
| LVSVi (mL/m^2^) | 39±12 | 40±10 | 38±14 | 0.61 |
| LVEF (mL/m^2^) | 73 (60-77) | 73 (68-78) | 71 (58-76) | 0.28 |

Normally distributed data displayed as mean ± SD. Non-normally distributed data shown as median (interquartile range) unless otherwise specified.

*Statistically significant with two-sided *P* <0.05.

CMR, cardiovascular magnetic resonance; RVEDVi, body surface area (BSA)-indexed right ventricular end-diastolic volume; RVESVi, BSA-indexed right ventricular end-systolic volume; RVSVi, BSA-indexed right ventricular stroke volume; RVEF, right ventricular ejection fraction; LVEDVi/LVESVi/LVSVi/LVEF, left ventricular measurements as per RV.
